# Facial soft tissue depth of a contemporary adult Greek population

**DOI:** 10.1007/s00414-024-03305-0

**Published:** 2024-08-10

**Authors:** Gülçin Coşkun, Marina Fasoula, Nikolaos Bontozoglou

**Affiliations:** 1 School of History, Classics & Archaeology, University of Edinburgh, William Robertson Wing, Teviot Place, Edinburgh, EH8 9AG UK; 2 Athens Medical Centre, Filadelfeos & Kefalariou 1, Kifisia, Athens 14562 Greece

**Keywords:** Forensic human identification, Forensic facial approximation, Facial soft tissue depth, Craniofacial identification, Hausdorff distance, Amira

## Abstract

Facial approximation is a technique that involves constructing the facial muscles and applying a suitable facial soft tissue depth (FSTD) dataset. To date, several FSTD studies have been conducted for varying population groups. This study aims to establish a FSTD dataset of an adult Greek population sample for the first time. The facial depths of subjects were measured on 100 head CT scans of 50 male and 50 female subjects aged from 18 to 99. The 3D head and skull models of subjects were segmented in Amira 6.1 by using histogram method. FSTDs were measured at 22 cranial landmarks (5 mid-sagittal, 17 bilateral). The FSTD dataset was generated by considering the age and sex of subjects. The impact of age and sex on the FSTD was limited. Slight inter-population depth variations were reported. Facial asymmetry calculated between the bilateral landmarks was insignificant for both male and female subjects.

## Introduction

The identification of deceased individuals has legal and ethical significance in a medico-legal context [1]. Establishing the identity of unknown individuals facilitates solving criminal cases (e.g., homicide and unexpected natural death), identifying the victims of natural disasters (e.g., earthquakes, hurricanes, tsunamis) and war crimes (e.g., genocide) [2–4]. From an ethical point of view, returning bodies to their loved ones mitigates the grief of families as well as concludes the legal issues such as the death certificate, inheritance, life insurance benefit and so on [5, 6].

Identification of skeletonised remains is achieved by one of the positive identification methods, such as deoxyribonucleic acid (DNA) analysis and the comparison of dental records depending on the preservation condition of the remains and the availability of the antemortem data of the person in question [4]. Yet, these positive identification methods are not feasible if there is no antemortem data available [4, 7]. In this event, presumptive or possible identification methods are used to narrow down possible identities by eliminating mismatching data. The presumptive identification method consists of the anthropological examination of skeletal remains and requires establishing the biological profile of an unknown individual [8]. This profiling includes age, sex, stature, ancestral background, skeletal variation/ abnormalities and congenital or pathology/trauma-related anomalies on bones [2, 9–13].

When positive and presumptive identification methods are insufficient in pointing an identity, the facial approximation of an unidentified individual is performed as a last resort to create a face that resembles the appearance of the deceased in life to reach individuals who might know the possible identity [14]. The method relies on the application of facial soft tissue depth (FSTD) and the creation of facial features (eyes, nose, mouth, ears, etc.) [13]. To date, several population-specific FSTD datasets have been set to create precise depth datasets for facial approximation [15–26]. People of Greek ancestry are one of the population groups for which a FSTD dataset has not been generated yet. Therefore, the aim of this study is to fill the gap in the field and establish a FSTD database for adult Greeks.

## Materials and methods

The material of this study consisted of the head CT scans of 100 subjects (50 male and 50 female), with an average age of 59 years old (ranging between 18 and 99 years). The subjects underwent CT scans for diagnostic purposes at the radiology department of Athens Medical Centre, Athens, Greece. Thus, the individuals were not exposed to radiation specifically for this study. All the subjects were assumed Greek based on their surnames. Any self-confirmation of ancestral background or genetic evidence was not collected.

### CT scan protocol, landmarks and FSTD measurement

The CT scans were taken using the Siemens SOMATOM^®^ Sensation 64 (Siemens AG, Forchheim, Germany). The images were recorded following the tube current of 400 mAs, at the tube voltage of 20 kV, with a slice thickness of 2.4 mm and a matrix of 512 × 512 pixels. The subjects were scanned when they were in the supine position. All the images were obtained in the DICOM format (Digital Imaging and Communications in Medicine). Along with the CT scans, the age and sex information of the subjects were obtained for the statistical analyses.

**Fig. 1:**
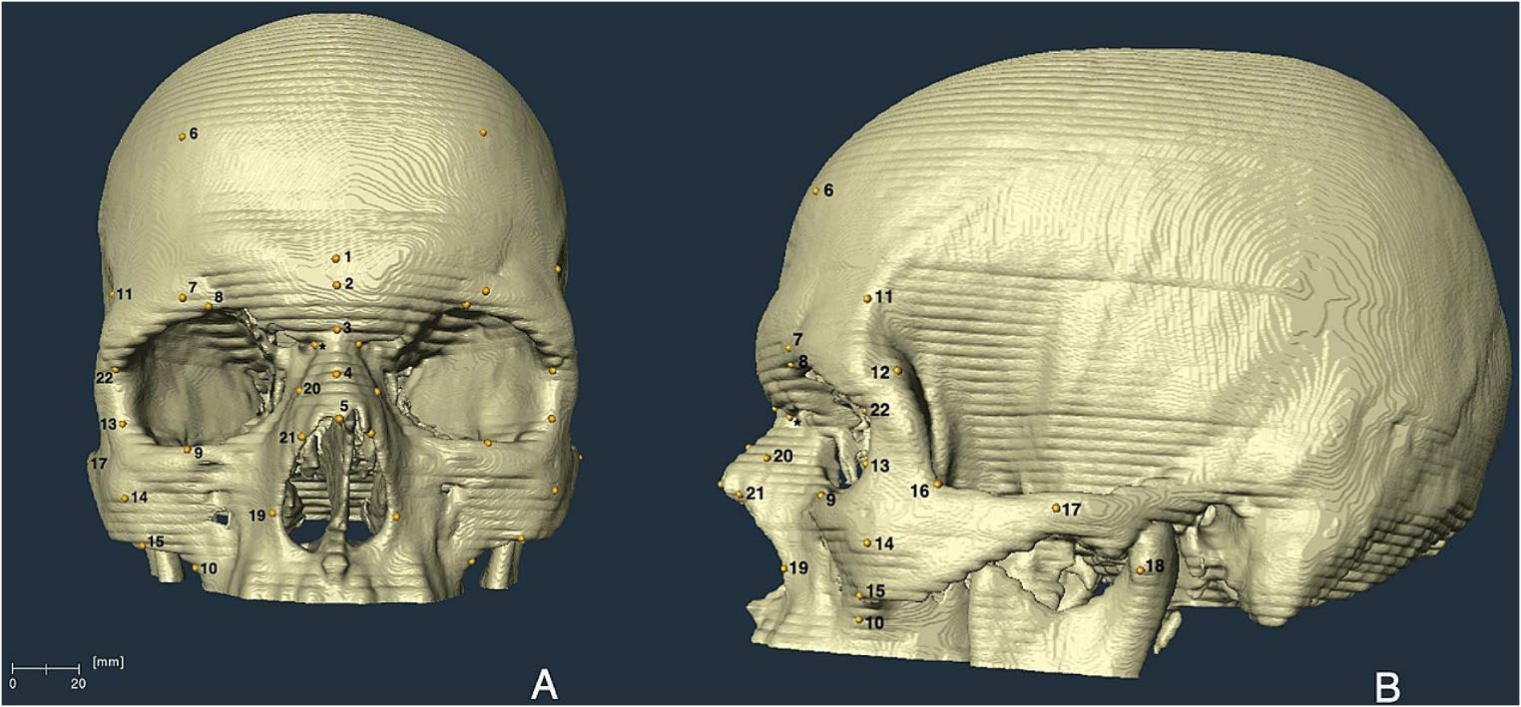
Anterior **(A)** and lateral **(B)** views of the 3D skull model, showing the cranial landmarks. (*): Nasomaxillofrontale. Due to the slice thickness of the CT scans (2.4 mm), horizontal lines appeared on the skull models

Commonly used 5 mid-sagittal and 17 bilateral cranial landmarks (Fig. 1; Table 1) were selected for this study to generate a comprehensive FSTD database. However, only those landmarks found on the upper portion of the skull were included in this study since the radiological examination did not require scanning the whole head, only the upper two-thirds of the skulls were scanned (from the top of the skull to the mastoid processes). Although the nasomaxillofrontale was identified on each skull to assist in locating the mid-nasomaxillare, FSTD from this landmark was not collected due to the obstruction caused by the eyelids in most of the cases.

**Fig. 2:**
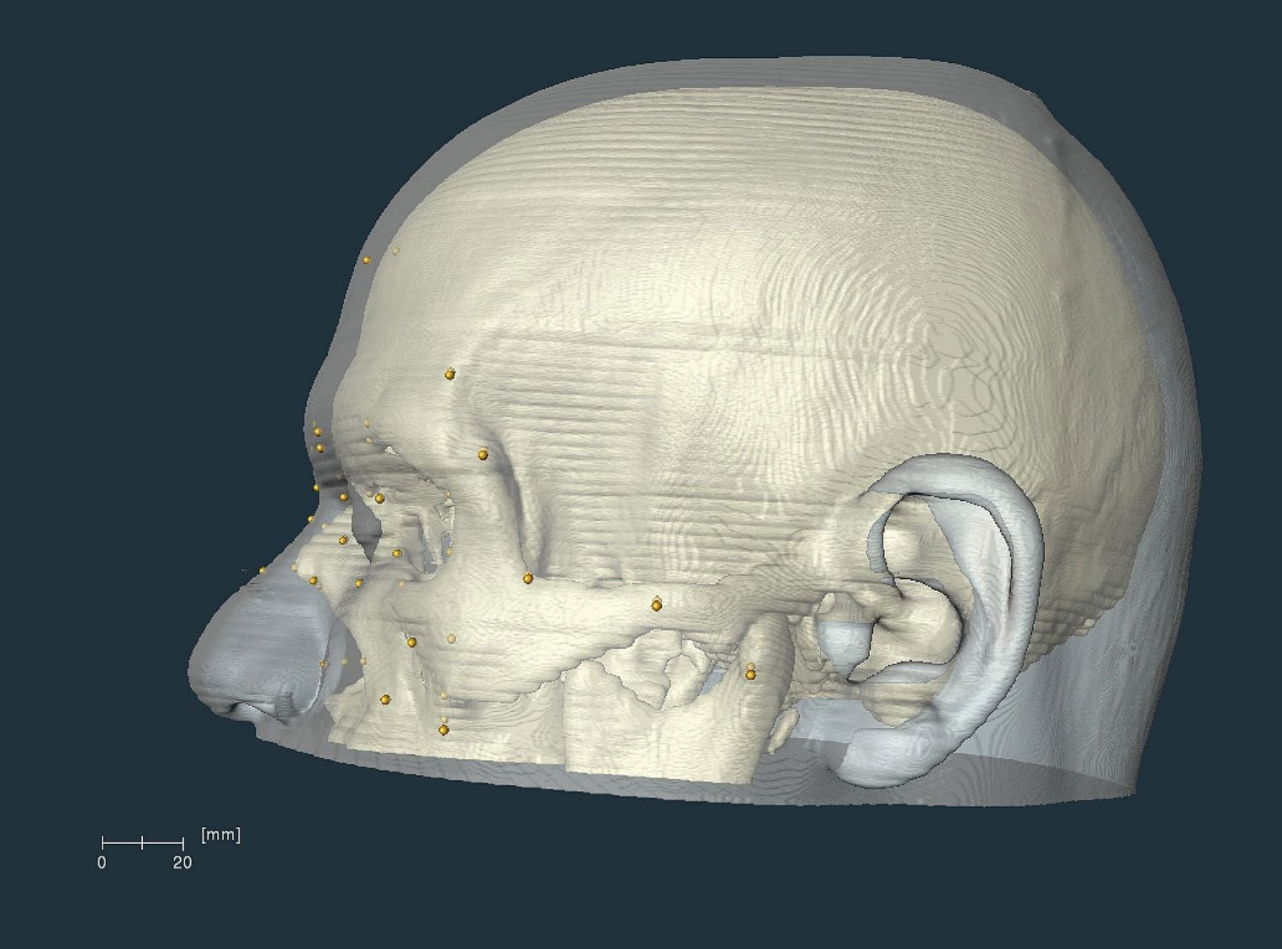
The superimposed 3D skull and head models in the sagittal plane

Before measuring the facial depths, the skulls and heads of the individuals were segmented using the histogram method [29] in AMIRA 6.1 (Thermo Fischer Scientific, Cleveland, OH, USA). Prior to taking measurements, the 3D skull models were positioned in the Frankfurt Horizontal plane (FHP), which implies that a plane passes through both right and left porions and both right and left orbitales [30]. When the skulls were still in the FHP, each cranial landmark was placed on each skull individually by following the description given in Table 1. This process was carried out in the sagittal, coronal and sagitto-coronal planes depending on the location of the landmarks (Table 1), as described by Guyomarc’h et al. [17]. Once all the cranial landmarks were placed, the 3D head and skull models were superimposed using the surface generation algorithm of Amira (Fig. 2). This algorithm provides visualising surfaces in different styles (e.g., solid, transparent, etc.). The head models were created using the transparent surface feature to ensure that the cranial landmarks could be easily identified and traced with precision (Fig. 2). The cephalometric landmarks were then placed on the 3D head models by perpendicularly tracing the cranial landmarks. Subsequently, the horizontal distance between the cranial and cephalometric landmarks was measured. All the measurements were taken by the first author.

**Table 1:** The definitions, abbreviations and synonyms of the cranial landmarks selected for the current study

Cranial Landmarks	Definition	Synonym
1. Supraglabella (Sg)	On the frontal bone, 10 mm superior to the glabella^20^	Ophyron^20^
2. Glabella (G)	Most anterior point between supraorbital ridges^18^	
3. Nasion (N)	The midpoint of the suture between the frontal and the two nasal bones^18^	
4. Mid-nasal (Mn)	On the internasal suture, midway between the nasion and rhinion^20^	
5. Rhinion (Rhi)	The anterior tip of the nasal bone^16^	End of nasals^17^
6. Frontal Eminence (Fe)	Place on the projections at both sides of the forehead^15^	
7. Supraorbital (So)	Most anterior point of the supraciliary arch in the axe of the centre of the orbit^20^	Superciliare^17^; superior eye orbit^17^ Mid-supraorbital^20^
8. Supraconchion (Sk)	Most superior point of the orbital rim^17^	Orbitale superius^17^
9. Orbitale (Or)	Most inferior point of the orbital margin^17^	Inferior eye orbit^17^
10. Sub-maxillar Curvature (Smc)	Most supero-medial point on the maxillary inflexion between the zygomaxillare and the ectomolare^17^	
11. Frontotemporale (Ft)	Most anterio-medial point of the linea temporalis superior^17^	
12. Frontomalare Temporale (Fmt)	Most posterior point of the zygo-frontal suture^17^	Mid-lateral orbit^17^; lateral eye orbit^17^
13. Ectoconchion (Ec)	The most lateral point at the lateral margin of orbit^19^	
14. Mid-zygomatic (Mz)	Lined up with the lateral border of the orbit on the centre of the zygomatic process^20^	Lateral orbit^20^; zygomatic malare^20^
15. Zygomaxillare (Zm)	Most inferior point on the zygo-maxillary suture^17^	
16. Jugale (Ju)	Most antero-inferior point on the posterior border of the zygomatic bone^17^	
17. Zygion (Zy)	Most lateral extent of the lateral surface of the zygomatic arch^17^	Root of zygoma^17^; Lateral zygomatic arch^17^; Zygomatic arch^27^
18. Condylion (Co)	Most lateral point of the glenoid process of the mandible^20^	Condylion laterale^20^
19. Alare (Al)	Most lateral point on the margin of the anterior nasal aperture^19^	Apertion^20^
20.Mid-nasomaxillare (Mnm)	Mid-point of the naso-maxillary suture between the nasomaxillare and the nasomaxillofrontale^17^	Lateral nasal^17^
21. Nasomaxillare (Nm)	Most inferior point of the naxo-maxillary suture on the nasal aperture^17^	
22. Frontomalare Orbitale (Fmo)	Point where the frontozygomatic suture crosses the inner orbital rim^20^	
23. Nasomaxillofrontale (Nmf)	Junction of the frontal, maxillary and lacrimal bones on the medial bone of the orbit^20^	Lateral Glabella^20^

Abbreviations, definitions and synonyms were obtained from the following publications: 15, 27, 16, 19, 17, 18, 20

### Accuracy assessment

Inaccurate segmentation of the 3D skull and head models might impact the accuracy of FSTD measurements. Therefore, before proceeding to the facial depth measurement process, the reproducibility and precision of the 3D models were tested by re-segmenting the head and skull models of randomly selected eight subjects at the five-week interval. The results were evaluated using Meshlab (Visual Computing Lab-ISTI-CNR, Pisa& Rome, Italy) by calculating the Hausdorff distance (HD), which is the longest distance between the two closest points on each 3D model pair when they are superimposed [31]. When HD equals zero, it indicates that the two models are identical. On the other hand, the HD values above 0.5 mm are considered a poor re-segmentation. Thus, the HD values that are close to zero suggest an accurate segmentation [35].

Additionally, the accuracy, repeatability and reproducibility of the measurements were calculated for intra- and inter-observer errors. The former was determined by the first author by re-measuring the FSTD of randomly selected subjects (*n* = 25) at a four-week interval. The latter, however, was measured by a second examiner who has no prior experience in identifying the cranial landmarks on 3D skull models and measuring FSTD. These measurements were re-collected on randomly selected 12 subjects at a four-week interval by following the same measurement protocol. The differences between the repeating measurements were determined by calculating the technical error of measurements (TEM) by applying the following formula:

$$\text{TEM}=\sqrt{\frac{{D}2}{2{N}}}$$

Where D is the difference between the first and the second measurements, N is the number of measurements re-taken [32, 33]. While the small TEM values are associated with the low TEM, the large mean values are an indication of the high TEM values. This positive correlation between the TEM values and the size of the measurements causes difficulty when two different sizes of measurements or variables are compared [34]. In order to tackle this problem, the TEM value is converted into the relative technical error measurements (rTEM) so as to indicate the error margin in percentage. This conversion is made by the formula given:

$$\text{r}\,\text{TEM}=\frac{{TEM}}{{VAV}}\times100$$

X 100

Where VAV is the variable average value, and it was calculated by computing the arithmetic mean value of the mean values of the first and second measurements [33]. The results lower than 5% are considered reliable [32, 34]. Lastly, the coefficient of reliability (R) was calculated to determine the portion of error-free measurements by applying the formula given below:

$$\text{R}=1-[\frac{{\left(TEM\right)}^{2}}{{S}^{2}}]$$]

Where S^2^ is the square of the absolute mean difference. The results of the R-value range between 1 and 0. The values lower than 0 are considered unreliable, whereas the R values that fall between 0.8 and 1 represent accurate results [32, 34].

### Statistical analysis

Data were analyzed using the Statistical Package for Social Sciences (SPSS, version 25.0). General descriptive analysis was performed for each landmark and mean facial soft tissue depths were calculated considering the age and sex categories of the individuals. Prior to performing statistical analyses, the individuals were divided into five age categories as follows: 18–34 years, 35–44 years, 45–54 years, 55–64 years and ≥ 65 years.

The significance level of < 0.05 was set for all the statistical tests. The distribution of normality was tested by using the *Shapiro-Wilk* test. The significance of the impact of age and sex on FSTD was tested by performing a multivariate analysis of variance (MANOVA). It should be noted that the impact of body mass index (BMI) on FSTD couldn’t be measured since the height and weight information of patients were not recorded at the hospital. A paired sample *t*-test was used to quantify the facial asymmetry for normally distributed data and the *Wilcoxon-signed rank* test for non-normally distributed data. The FSTDs of this study were compared with the reported mean FSTD values of five populations [17, 19, 22, 23, 37] which have a similar measurement protocol. The comparisons were performed by using the one sample *t*-test for normally distributed data and the *Wilcoxon one-sample signed-ranked* test was utilised for non-normally distributed data. Bootstrap 1000 was used for all statistical tests in order to explain possible biases that occurred due to the small sample size.

## Results

### Accuracy assessment of skull and head segmentations

The results showed that the head and skull models of this study were reproduced with good precision. All the HD values of the head models were lower than 0.5 mm, ranging between 0.01 and 0.15 mm (Table 2). The lowest HD value (high precision) was recorded from Head 1. The same HD values were reported from Heads 5, 6 and 8, which was 0.02 mm. The highest HD score (low precision) was reported from Head 3 with 0.15 mm, which was still significantly below 0.5 mm [35]. The HD values of the skull models were slightly higher than those of the head models (Table 2). The only HD value that exceeded 0.5 mm was the Skull 4 with 0.63 mm.

**Table 2:** Hausdorff distances (HD) calculated for the skull and head models (measurements are in mm)

Skull/Head No	Head	Skull
	Hausdorff distance	Hausdorff distance
1	0.01	0.04
2	0.12	0.36
3	0.15	0.36
4	0.11	0.63
5	0.02	0.14
6	0.02	0.08
7	0.08	0.01
8	0.02	0.03

HD values suggested that the head models were re-segmented with higher precision in comparison with the skull re-segmentations. This difference was due to the contrast between neighbouring voxels. When segmenting the head models, the contrast between the voxels of air (black) and the head (grey) was high. Therefore, the colour difference between the voxels was more distinguishable. On the other hand, differentiating the voxels belonging to the skull (light grey/white) from those of the head (grey) was rather challenging due to the partial volume effect [36]. Hence, the skull re-segmentations were completed with less precision compared to the head models.

### Intra- and inter-observer error

The results of the intra-observer error assessment showed that all the measurements were within the acceptable accuracy range. The absolute TEM values were generally low, fluctuating between 0.10 mm and 1.05 mm (Table 3). The highest FSTD values were generally collected from the cheek region. Since there is a positive correlation between the high-depth measurements and high TEM values, obtaining high TEM values from the cheek region was anticipated. In order to solve this, the TEM values were converted into the rTEM values. The rTEM values were at or below 5%, ranging between 1.6% and 5%. The highest rTEM value was obtained from the mid-nasal (5%), yet it was still considered a repeatable measurement [32]. The R values were, however, close to 1, ranging between 0.84 and 0.99 mm. The lowest R values were obtained for the left and right sub-maxillar curvature (0.88 and 0.84 mm, respectively). However, they were still within the acceptable accuracy range [32].

The absolute TEM values calculated for the inter-observer error varied between 0.19 and 1.45 mm. The high absolute TEM values were calculated for the left zygomaxillare, left and right sub-maxillar curvature, right condylion and right nasomaxillare. The rTEM values showed significant variation compared to those of the intra-observer error. The rTEM percentages of 22 landmarks were above 5% (Table 3). Therefore, the repeated measurements of the 22 landmarks were considered unreliable for the inter-observer error. On the other hand, the rTEM scores of the 17 landmarks were found to be reliable. The R values of inter-observer error were mostly within the 0.80–1 mm accuracy range. Only the mid-nasal, right sub-maxillar curvature, left zygomaxillare and right nasomaxillare were lower than 0.80 mm. These results suggested that experience impacts the accuracy of FSTD measurements.

**Table 3:** TEM, rTEM and coefficient of reliability (R) results reveal intra- and inter-observer errors calculated for the 3D measurement method

	Intra-Observer Error				Inter-Observer Error				
Landmarks	n	TEM (mm)	rTEM (%)	R	n	TEM (mm)	rTEM (%)	R	
Sg	25	0.20	3.5	0.97	12	0.27	4.53	0.95	
G	25	0.10	1.8	0.99	12	0.20	3.18	0.96	
N	25	0.11	1.6	0.99	12	0.23	3.48	0.96	
Mn	25	0.14	5.0	0.97	12	0.47	18.35	0.63	
Rhi	25	0.16	4.7	0.94	12	0.23	6.64	0.93	
FeL	25	0.21	3.3	0.97	12	0.43	6.36	0.94	
FeR	25	0.21	3.3	0.97	12	0.66	9.47	0.93	
SoL	25	0.26	3.0	0.97	12	0.38	5.00	0.95	
SoR	25	0.17	1.9	0.98	12	0.48	6.05	0.93	
SkL	25	0.29	3.2	0.98	12	0.89	9.76	0.84	
SkR	25	0.37	4.2	0.95	12	0.47	5.07	0.91	
OrL	25	0.14	2.1	0.99	12	0.32	4.65	0.98	
OrR	25	0.20	2.9	0.99	12	0.33	4.86	0.97	
SmcL	25	0.94	3.4	0.88	12	1.08	3.98	0.80	
SmcR	24	1.05	3.8	0.84	12	1.06	3.78	0.66	
FtL	25	0.29	4.5	0.96	12	0.40	6.60	0.92	
FtR	25	0.27	4.2	0.98	12	0.48	6.73	0.95	
FmtL	25	0.22	3.2	0.98	12	0.61	9.70	0.80	
FmtR	25	0.32	3.8	0.97	12	0.64	9.47	0.89	
EcL	25	0.26	2.9	0.99	12	0.81	9.67	0.91	
EcR	25	0.22	2.6	0.99	12	0.96	11.39	0.86	
MzL	25	0.15	1.6	0.99	12	0.21	2.18	0.98	
MzR	25	0.23	2.2	0.98	12	0.30	2.97	0.97	
ZmL	25	0.64	4.5	0.90	12	1.45	8.69	0.58	
ZmR	25	0.73	4.7	0.90	12	0.85	4.98	0.84	
JuL	25	0.48	4.8	0.93	12	0.26	2.62	0.98	
JuR	25	0.32	2.9	0.96	12	0.44	4.15	0.94	
ZyL	25	0.30	3.1	0.98	12	0.22	2.22	0.99	
ZyR	25	0.22	2.3	0.99	12	0.23	2.36	0.99	
CoL	25	0.41	2.2	0.98	12	0.19	0.97	0.99	
CoR	25	0.40	2.2	0.98	12	1.12	5.88	0.93	
AlL	25	0.27	2.8	0.98	12	0.59	5.93	0.95	
AlR	25	0.30	3.1	0.98	12	0.42	4.62	0.96	
MnmL	25	0.15	4.8	0.98	12	0.31	10.88	0.94	
MnmR	24	0.15	4.8	0.98	12	0.44	13.91	0.90	
NmL	25	0.25	4.5	0.98	12	0.78	14.86	0.84	
NmR	25	0.25	4.2	0.98	12	1.21	22.94	0.73	
FmoL	25	0.24	2.5	0.99	12	0.85	9.97	0.93	
FmoR	25	0.38	4.0	0.98	12	0.95	11.56	0.91	

### FSTD dataset

Tables 4, 5, 6, 7 and 8 show the descriptive statistics for the FSTDs measured at each anatomic landmark, considering the age groups (18–34, 35–44, 45–54, 55–64 and ≥ 65 years) of both males and females.

**Table 4:** Facial soft tissue thicknesses of the male and female individuals that were obtained using the 3D measurement method for the 18–34 years age group (measurements in mm)

Landmarks	Male (*n* = 8)					Female (*n* = 8)				
	*n*	$\:\stackrel{-}{\varvec{\upchi\:}}$	*σ*	Min	Max	*n*	$\:\stackrel{-}{\varvec{\upchi\:}}$	*σ*	Min	Max
Sg	8	6.48	2.52	4.70	8.62	7	4.79	0.72	4.00	6.14
G	8	5.99	2.02	4.56	7.73	8	4.85	0.56	4.20	8.69
N	8	7.30	0.54	5.80	9.65	8	5.57	1.08	4.36	9.98
Mn	5	4.17	1.07	2.02	4.93	7	2.39	0.71	1.55	3.33
Rhi	6	3.09	0.17	2.09	4.63	8	2.57	0.64	1.97	3.24
FeL	6	6.48	0.48	6.14	6.97	7	5.93	0.90	4.85	6.95
FeR	6	5.96	1.22	5.10	7.15	7	6.57	1.89	4.39	7.76
SoL	8	8.25	0.28	7.69	11.53	8	6.77	0.66	6.01	12.64
SoR	8	8.11	0.25	7.35	10.90	8	6.56	1.30	5.14	10.65
SkL	8	9.54	3.01	7.41	11.67	8	6.66	0.62	5.97	12.34
SkR	8	8.78	2.26	7.18	10.64	8	6.05	1.00	4.99	11.75
OrL	8	5.74	3.21	3.47	12.04	8	4.87	2.28	3.31	8.64
OrR	8	6.44	3.55	3.93	9.84	8	5.16	1.79	3.95	8.12
SmcL	7	22.71	2.11	21.22	35.71	6	23.63	2.63	21.24	28.93
SmcR	6	25.54	6.00	21.30	34.00	6	25.94	2.24	23.78	29.82
FtL	8	5.76	0.58	4.43	9.97	8	5.09	0.80	4.37	7.33
FtR	8	5.94	0.73	4.26	9.11	8	4.99	0.35	4.64	8.37
FmtL	8	7.70	2.07	5.32	10.24	8	5.35	0.93	4.30	9.31
FmtR	8	9.92	0.95	6.16	11.25	8	6.70	2.34	4.06	10.00
EcL	8	6.48	0.01	4.11	14.90	8	10.85	6.72	3.64	16.95
EcR	6	5.64	0.70	5.14	16.22	8	7.72	4.72	2.32	11.06
MzL	7	7.14	0.83	6.21	13.25	8	7.34	0.96	6.26	11.08
MzR	7	7.91	0.59	7.49	12.18	8	8.37	0.76	7.89	12.27
ZmL	7	12.07	0.54	11.03	20.07	8	11.46	2.36	9.83	17.75
ZmR	7	13.90	1.83	12.60	17.83	8	12.40	2.39	10.94	19.60
JuL	7	8.63	0.51	7.06	13.81	8	7.79	1.26	7.04	12.64
JuR	7	9.11	0.22	7.73	12.27	8	8.83	1.34	7.65	11.51
ZyL	8	7.55	0.13	5.16	13.97	8	7.86	0.98	6.93	12.43
ZyR	7	7.49	0.74	5.34	12.42	8	8.11	0.76	7.32	13.01
CoL	8	16.77	1.17	13.46	29.90	8	13.70	2.27	11.88	21.38
CoR	8	16.15	2.34	13.14	28.86	8	12.98	1.48	11.27	20.37
AlL	7	9.03	1.88	7.70	14.52	7	7.68	0.88	5.92	12.25
AlR	7	10.44	1.51	7.48	12.27	7	7.94	1.49	6.09	13.10
MnmL	6	2.57	0.01	2.56	4.79	8	2.52	0.31	2.21	5.00
MnmR	6	2.60	0.22	2.44	4.81	8	2.52	0.56	2.05	4.76
NmL	6	4.39	0.26	4.20	9.46	7	4.16	0.36	3.52	5.99
NmR	6	5.07	1.13	4.27	9.32	5	4.65	0.36	2.95	5.04
FmoL	8	6.89	1.57	5.78	14.25	8	10.15	2.65	6.94	14.49
FmoR	8	6.16	0.21	6.01	14.64	8	8.92	2.71	5.61	14.13

*n* = number of samples, $\:\stackrel{-}{\varvec{\upchi\:}}$= mean, *σ* = standard deviation, Min = minimum, Max = maximum

**Table 5:** Facial soft tissue thicknesses of the male and female individuals that were obtained using the 3D measurement method for the 35–44 years age group (measurements in mm)

	Male (*n* = 5)					Female (*n* = 5)				
Landmarks	*n*	$\:\stackrel{-}{\varvec{\upchi\:}}$	*σ*	Min	Max	*n*	$\:\stackrel{-}{\varvec{\upchi\:}}$	*σ*	Min	Max
Sg	5	7.62	1.41	4.00	8.61	5	5.49	1.65	4.32	6.66
G	5	6.89	2.24	4.37	8.97	5	5.64	1.41	4.64	6.67
N	5	8.79	1.18	5.51	10.95	5	6.11	2.09	4.63	7.98
Mn	5	3.37	1.21	2.46	6.67	5	2.80	0.65	1.80	3.26
Rhi	5	4.22	0.59	2.40	4.63	5	2.47	0.59	2.05	3.05
FeL	5	7.64	0.83	4.60	8.49	5	6.61	2.06	5.15	8.06
FeR	5	6.60	0.43	4.18	9.16	5	5.49	1.32	4.56	7.42
SoL	5	11.48	1.44	9.16	12.50	5	7.22	0.25	6.37	9.24
SoR	5	10.94	0.37	9.71	11.70	5	7.07	0.93	6.41	8.60
SkL	5	10.92	2.26	8.87	14.53	5	6.92	0.71	6.41	8.25
SkR	4	10.74	1.39	9.59	11.72	5	6.54	0.69	6.05	8.12
OrL	5	9.19	3.15	3.96	11.42	5	5.55	0.08	5.20	6.83
OrR	5	8.97	2.88	4.20	11.01	5	4.91	1.73	3.68	7.47
SmcL	3	27.67	1.79	23.47	28.93	4	27.43	3.68	24.83	30.03
SmcR	3	29.77	4.70	26.44	33.09	4	27.81	4.56	24.58	31.03
FtL	5	7.55	1.67	3.43	8.73	5	5.28	1.85	3.97	6.91
FtR	5	7.79	1.48	3.10	8.83	5	4.85	0.57	3.97	7.99
FmtL	5	8.42	1.00	7.29	9.13	5	5.82	2.04	3.60	10.03
FmtR	5	8.67	2.35	6.52	10.33	5	6.06	2.89	4.01	11.52
EcL	5	11.47	1.13	3.26	14.40	5	6.50	1.36	4.49	12.67
EcR	4	9.33	4.24	4.55	12.32	5	6.21	1.70	2.32	12.60
MzL	3	9.57	2.31	7.93	11.20	5	9.88	1.53	8.80	11.98
MzR	3	10.27	1.24	9.39	11.33	5	9.71	1.32	8.77	13.32
ZmL	3	14.93	0.23	14.76	15.85	5	13.34	2.18	11.79	16.35
ZmR	3	16.96	0.27	16.77	17.57	5	13.60	1.47	12.56	21.20
JuL	5	9.29	0.44	7.76	9.62	5	8.62	3.37	6.23	12.96
JuR	5	9.86	1.25	7.45	10.74	5	9.55	3.22	7.27	13.22
ZyL	5	9.89	0.70	7.60	10.38	5	9.54	5.91	5.36	13.72
ZyR	5	10.85	1.46	6.93	11.88	5	9.64	5.15	6.00	13.28
CoL	5	20.13	3.99	16.57	23.01	5	13.98	7.61	8.60	25.82
CoR	5	19.38	4.84	13.82	25.71	5	13.22	6.00	8.98	25.17
AlL	3	10.52	1.08	8.90	11.28	5	8.60	0.38	7.04	13.03
AlR	3	9.04	0.49	8.69	9.84	5	8.00	1.79	6.73	11.56
MnmL	5	3.56	0.59	2.50	3.98	5	2.43	0.04	1.81	4.89
MnmR	5	4.01	1.42	2.41	5.01	5	2.33	0.13	2.23	4.94
NmL	5	5.88	0.35	3.74	9.18	4	4.59	0.18	4.33	5.65
NmR	5	6.69	1.22	4.65	8.39	3	4.49	0.46	4.16	4.83
FmoL	5	15.80	1.18	4.98	16.63	5	9.59	2.28	5.22	11.41
FmoR	5	14.74	1.07	6.39	17.87	5	8.33	1.83	5.30	11.29

*n* = number of samples, $\:\stackrel{-}{\varvec{\upchi\:}}$= mean, *σ* = standard deviation, Min = minimum, Max = maximum

**Table 6:** Facial soft tissue thicknesses of the male and female individuals that were obtained using the 3D measurement method for the 45–54 years age group (measurements in mm)

Landmarks	Male (*n* = 9)					Female (*n* = 6)				
	*n*	$\:\stackrel{-}{\varvec{\upchi\:}}$	*σ*	Min	Max	*n*	$\:\stackrel{-}{\varvec{\upchi\:}}$	*σ*	Min	Max
Sg	9	5.19	0.46	4.74	8.21	6	6.01	0.74	5.49	7.75
G	9	5.35	0.39	4.29	8.70	6	6.01	0.78	5.46	7.69
N	9	6.95	1.09	4.98	10.71	6	7.28	1.65	6.11	8.45
Mn	8	3.14	0.70	1.90	4.13	5	2.98	1.05	2.24	3.74
Rhi	8	3.53	0.77	2.32	4.57	5	2.91	0.40	2.04	3.27
FeL	8	5.97	1.52	4.28	7.52	6	5.48	1.36	4.52	6.74
FeR	9	5.89	1.54	4.11	8.24	6	5.51	1.80	4.23	7.80
SoL	8	9.25	0.26	6.94	12.19	6	8.33	0.69	7.10	10.25
SoR	8	8.81	0.76	7.70	12.91	6	8.20	1.12	7.41	9.58
SkL	9	9.83	0.85	6.06	14.20	6	8.51	0.71	7.58	10.02
SkR	8	10.02	0.97	7.64	13.50	6	9.15	0.64	8.27	9.93
OrL	9	8.37	2.57	2.65	12.08	6	5.52	0.12	3.57	7.84
OrR	9	7.62	2.59	3.33	11.55	6	5.67	1.00	3.57	9.54
SmcL	7	27.73	0.73	26.90	36.07	5	25.62	0.62	25.18	31.31
SmcR	7	29.11	1.51	27.59	35.24	5	26.36	0.52	24.99	29.16
FtL	9	5.78	1.41	4.78	8.84	6	6.42	1.03	4.01	9.59
FtR	9	5.49	1.22	4.13	7.65	6	6.76	1.54	3.90	8.59
FmtL	9	8.82	1.61	6.58	10.51	6	6.69	3.64	4.11	9.26
FmtR	9	8.62	1.54	6.51	11.92	6	7.40	3.94	4.61	10.41
EcL	9	13.48	2.29	5.56	19.16	6	10.03	4.38	5.63	13.12
EcR	9	12.19	0.97	6.21	16.44	6	9.08	3.19	5.91	11.33
MzL	6	8.02	1.14	6.92	9.99	5	8.78	0.47	8.44	12.05
MzR	6	8.84	1.67	7.29	10.61	5	9.19	0.74	8.66	12.43
ZmL	6	13.26	1.18	11.90	18.44	5	13.00	0.86	12.39	16.85
ZmR	6	15.78	0.84	14.81	17.26	5	14.08	0.13	13.98	19.33
JuL	9	10.45	0.98	7.95	13.81	6	9.88	0.18	9.75	14.19
JuR	9	10.99	0.44	8.74	12.87	6	10.40	0.01	8.78	14.14
ZyL	8	8.61	0.51	6.80	12.58	6	9.15	0.99	8.40	13.65
ZyR	9	10.11	0.58	7.55	14.29	6	9.91	1.05	9.17	13.31
CoL	9	19.10	3.51	16.72	25.83	6	16.20	2.40	14.50	21.42
CoR	9	20.52	1.22	12.25	25.90	6	15.20	0.44	14.89	22.85
AlL	7	9.38	1.68	7.44	13.64	5	7.69	0.01	6.10	14.48
AlR	7	9.75	0.30	9.50	11.29	5	8.29	0.66	5.34	11.54
MnmL	9	2.84	0.31	2.33	3.71	6	2.52	0.08	2.02	3.76
MnmR	9	3.39	0.32	2.72	4.04	6	2.49	0.37	2.13	3.33
NmL	8	6.08	1.13	3.73	7.18	5	4.43	0.20	3.11	5.48
NmR	9	6.08	0.83	3.35	9.15	3	4.41	0.06	2.80	4.45
FmoL	9	14.41	0.73	4.96	18.84	6	9.62	4.97	6.10	13.26
FmoR	9	14.49	3.45	5.73	17.74	6	9.46	4.60	6.20	14.13

*n* = number of samples, $\:\stackrel{-}{\varvec{\upchi\:}}$= mean, *σ* = standard deviation, Min = minimum, Max = maximum

**Table 7:** Facial soft tissue thicknesses of the male and female individuals that were obtained using the 3D measurement method for the 55–64 years age group (measurements in mm)

Landmarks	Male (*n* = 7)					Female (*n* = 6)				
	*n*	$\:\stackrel{-}{\varvec{\upchi\:}}$	*σ*	Min	Max	*n*	$\:\stackrel{-}{\varvec{\upchi\:}}$	*σ*	Min	Max
Sg	7	5.72	1.23	4.34	7.77	6	6.66	0.91	6.17	8.28
G	7	5.42	1.45	3.99	7.22	6	6.60	0.37	6.31	7.13
N	7	6.50	1.15	5.82	10.05	6	7.38	1.20	6.57	9.34
Mn	6	2.79	1.05	2.10	4.61	5	2.79	0.82	1.95	3.85
Rhi	6	3.58	0.22	1.81	3.77	5	2.55	0.49	2.07	2.89
FeL	6	6.58	0.41	3.96	6.94	5	5.39	0.18	5.26	8.45
FeR	6	6.46	0.82	4.94	7.23	6	6.41	0.40	5.16	7.61
SoL	7	10.22	0.84	7.14	12.17	5	9.32	0.93	8.66	11.19
SoR	7	9.92	0.61	8.38	13.03	6	7.34	2.02	5.91	11.22
SkL	7	10.02	1.33	8.09	12.75	5	10.01	0.47	9.68	11.76
SkR	7	10.89	1.46	8.85	13.21	6	7.73	2.90	5.68	10.96
OrL	7	6.75	0.70	4.05	7.47	5	7.43	1.72	6.21	9.28
OrR	7	8.16	1.89	4.31	9.99	6	7.40	0.24	6.76	10.42
SmcL	5	29.99	3.90	24.18	33.83	5	28.38	2.52	26.59	30.90
SmcR	6	30.50	0.81	25.81	31.41	4	30.78	0.01	30.77	33.20
FtL	7	6.09	0.83	4.88	7.95	5	7.72	1.59	5.34	9.29
FtR	7	6.36	1.29	5.09	8.15	6	7.07	0.51	5.84	7.07
FmtL	7	6.81	0.86	5.20	8.87	5	8.15	1.03	7.38	9.84
FmtR	7	8.32	1.03	5.40	11.16	6	9.42	0.97	7.79	10.19
EcL	7	12.26	1.34	5.48	13.45	5	10.63	2.39	6.30	15.59
EcR	7	11.80	0.96	6.46	12.79	6	10.99	3.56	9.38	17.59
MzL	7	11.01	1.56	6.49	12.26	5	9.87	1.42	9.87	12.69
MzR	7	11.17	0.59	8.25	11.82	5	10.53	1.54	10.53	14.16
ZmL	6	14.93	1.82	11.30	16.00	5	14.49	1.37	14.49	17.59
ZmR	6	16.42	1.13	14.21	19.05	5	17.23	0.73	16.57	19.01
JuL	7	10.31	1.47	6.48	11.78	6	12.75	0.70	11.09	12.89
JuR	7	10.69	1.34	7.40	11.84	6	12.92	0.53	11.67	12.92
ZyL	7	10.13	2.52	6.08	12.82	6	9.66	1.06	9.66	12.17
ZyR	7	10.53	2.30	6.26	13.14	6	9.62	1.73	9.62	14.20
CoL	7	21.49	3.01	18.07	23.76	6	19.57	3.04	15.94	23.56
CoR	7	22.25	3.81	16.41	24.66	6	19.87	2.80	16.29	24.02
AlL	6	8.77	1.05	7.26	9.61	5	9.08	3.17	7.50	14.76
AlR	6	9.45	1.88	7.57	11.33	5	7.93	1.44	7.93	11.89
MnmL	7	3.10	1.14	1.92	4.42	4	3.07	0.45	2.43	3.07
MnmR	7	3.40	1.02	1.62	4.56	6	3.11	0.05	1.83	3.11
NmL	7	5.75	0.70	3.78	6.35	4	5.81	0.31	3.57	5.81
NmR	7	6.84	0.62	4.33	7.53	6	5.67	1.44	2.64	5.67
FmoL	7	13.49	1.52	8.97	14.89	5	13.40	6.00	4.91	15.39
FmoR	7	11.84	3.00	9.86	15.29	6	10.46	3.26	5.85	17.63

*n* = number of samples, $\:\stackrel{-}{\varvec{\upchi\:}}$= mean, *σ* = standard deviation, Min = minimum, Max = maximum

**Table 8:** Facial soft tissue thicknesses of the male and female individuals that were obtained using the 3D measurement method for the ≥ 65 years age group (measurements in mm)

Landmarks	Male (*n* = 21)					Female (*n* = 25)				
	*n*	$\:\stackrel{-}{\varvec{\upchi\:}}$	*σ*	Min	Max	*n*	$\:\stackrel{-}{\varvec{\upchi\:}}$	*σ*	*Min*	*Max*
Sg	21	6.10	1.20	3.11	9.46	24	5.33	1.04	4.39	8.04
G	20	5.88	1.00	3.54	9.23	24	5.66	0.64	3.86	7.61
N	21	7.36	1.53	4.66	11.02	25	5.16	0.74	4.27	9.69
Mn	17	2.96	0.61	1.98	4.95	22	2.15	0.23	1.73	4.01
Rhi	17	3.42	0.58	2.29	4.21	21	2.60	0.15	2.01	3.29
FeL	18	6.46	0.93	4.37	8.50	23	4.78	1.03	3.78	7.72
FeR	19	5.75	1.02	3.56	8.38	25	5.35	0.96	3.28	8.26
SoL	19	9.06	1.57	6.13	14.11	24	8.78	2.07	5.88	12.04
SoR	21	8.41	1.04	7.15	13.82	25	8.43	1.95	5.00	11.15
SkL	21	8.91	1.56	4.84	15.53	24	8.25	1.92	5.60	12.28
SkR	20	8.89	1.73	6.62	14.02	24	7.62	2.13	4.83	11.49
OrL	21	6.37	2.56	2.93	12.58	23	5.27	0.68	3.91	9.88
OrR	21	6.65	1.85	2.53	12.03	24	5.29	1.23	3.92	10.21
SmcL	18	25.16	2.56	21.09	36.11	16	28.76	1.76	22.11	32.94
SmcR	17	26.09	3.45	22.62	36.16	17	27.82	2.63	24.79	33.61
FtL	21	6.25	1.09	3.96	9.27	24	5.66	2.08	3.23	8.73
FtR	19	5.66	1.05	3.63	8.45	25	5.76	1.43	2.82	7.90
FmtL	21	6.00	1.66	3.74	10.54	24	4.72	0.53	3.89	12.73
FmtR	19	6.62	2.23	3.45	9.94	25	5.63	0.73	3.70	12.69
EcL	20	11.24	1.61	3.67	19.03	24	10.10	3.56	2.53	15.11
EcR	19	10.51	2.33	5.80	17.77	25	10.22	2.75	2.08	16.36
MzL	20	7.99	1.27	5.68	12.60	23	9.38	1.46	7.50	12.60
MzR	18	8.46	1.32	5.52	13.38	23	10.48	0.93	7.39	12.99
ZmL	19	12.20	1.80	9.25	18.89	23	12.32	1.25	9.66	18.01
ZmR	14	14.65	2.35	11.69	18.08	21	14.72	1.09	10.08	20.11
JuL	21	8.24	1.78	5.01	13.04	24	10.05	2.44	7.67	14.22
JuR	20	8.07	1.24	5.91	14.05	25	10.30	1.80	6.52	14.84
ZyL	21	7.14	1.29	5.45	12.89	24	9.17	1.45	5.44	15.72
ZyR	21	6.98	0.83	5.27	14.77	25	9.60	1.28	3.81	16.16
CoL	21	17.30	2.50	11.72	28.43	24	15.96	1.54	9.61	22.21
CoR	21	16.81	0.90	12.48	25.86	24	15.18	1.64	7.04	25.98
AlL	19	10.47	2.26	5.33	13.37	21	9.53	3.26	5.36	14.00
AlR	16	10.52	1.28	5.91	14.00	20	8.16	2.05	5.96	14.47
MnmL	19	2.80	0.73	2.01	5.19	22	2.11	0.39	1.47	4.33
MnmR	18	3.13	0.86	2.08	4.61	25	2.11	0.38	1.35	5.00
NmL	19	5.07	1.05	3.75	9.85	14	4.66	0.73	2.06	5.67
NmR	17	5.76	1.12	3.61	8.80	16	4.02	0.68	2.38	5.57
FmoL	21	10.14	3.33	3.77	23.07	24	8.96	3.98	2.51	15.98
FmoR	20	9.86	2.90	2.46	17.25	25	8.01	4.88	2.09	15.37

*n* = number of samples, $\:\stackrel{-}{\varvec{\upchi\:}}$= mean, *σ* = standard deviation, Min = minimum, Max = maximum

### Impact of age and sex variables

**Fig. 3:**
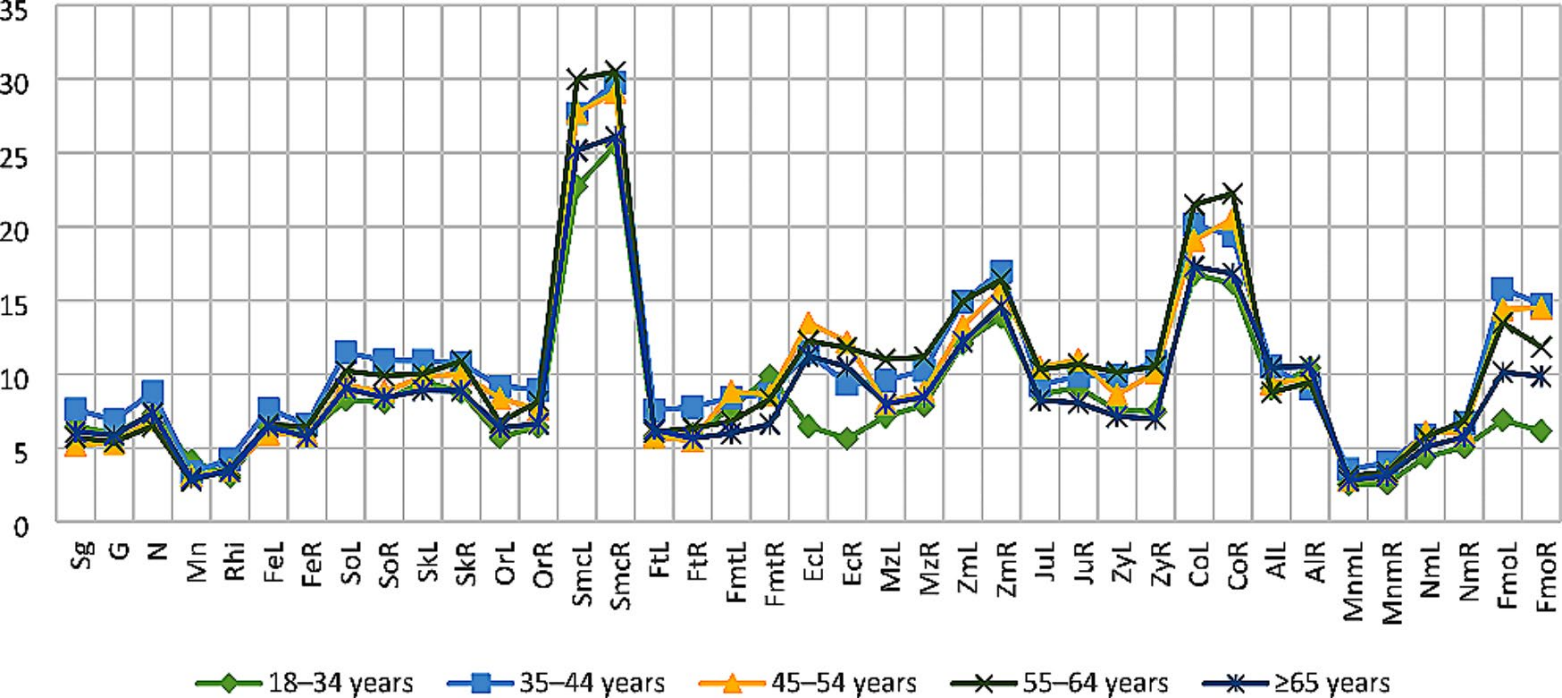
The line graph summarises the mean FSTDs of male subjects, including all five age groups

**Fig. 4:**
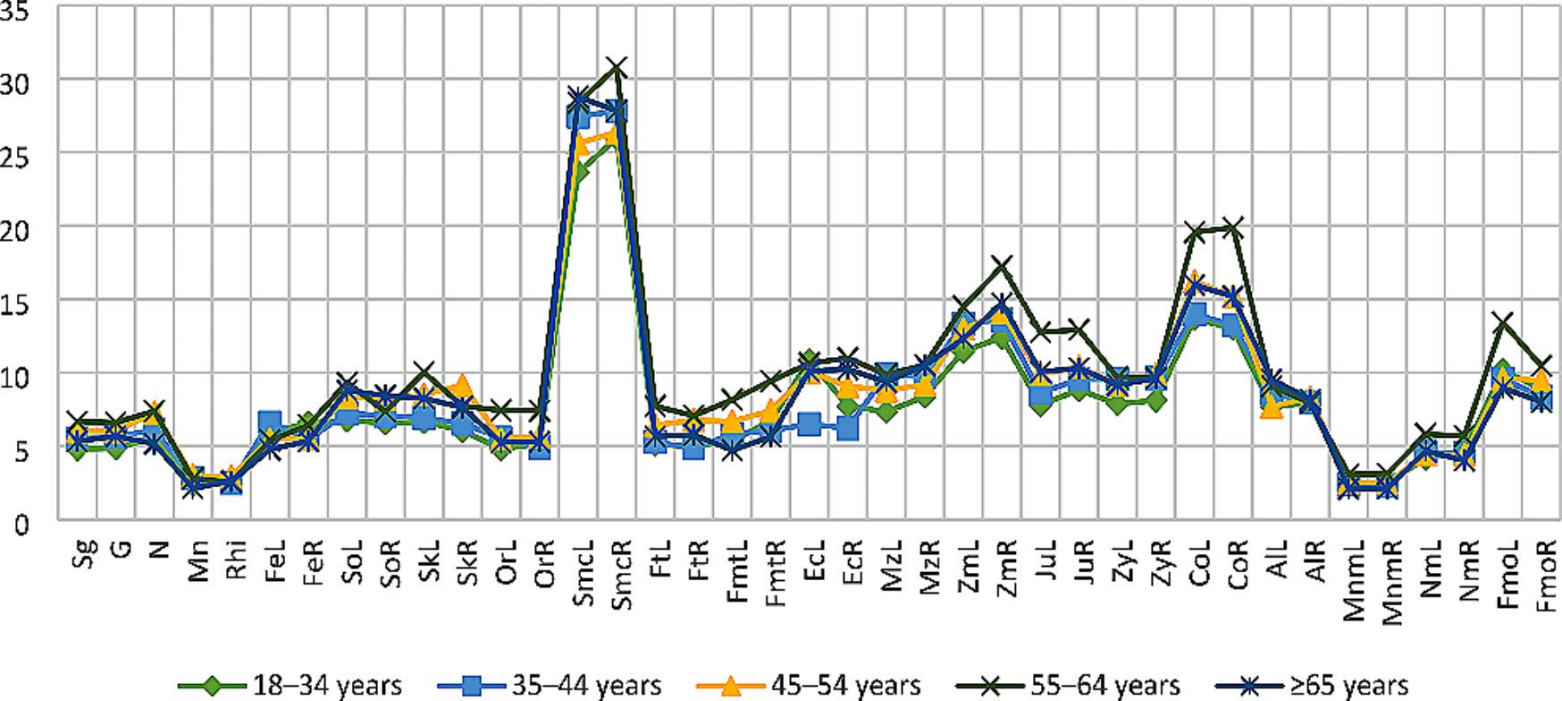
The line graph summarises the mean FSTDs of female subjects, including all five age groups

The p-values of the age and sex variables and the two factor-between-subject effects are presented in Table 9. The mean depth values between the age groups varied considerably in the cheek and orbital regions for both males and females (Figs. 3 and 4). However, only the left orbitale, left mid-zygomatic and right condylion were found to be influenced by increasing age at the statistically significant level (*p* < 0.05). The mean FSTDs of females slightly increased with age. The largest depth values were generally reported from the 55–64 years age group. After this age group, the depth scores decreased again. On the contrary in females, there was not a specific age-related increasing or decreasing pattern in males.

The mean depth differences indicated that the FSTDs of males were slightly higher than those of females. Only the cheek region tends to be thicker in females, especially the submandibular curvature and mid-zygomatic. Despite the depth differences obtained between males and females, only at the left orbitale and right zygomaxillare was sexually dimorphic at the statistically significant level (*p* < 0.05). Similarly, the combined impact of age and sex variables was limited on the FSTDs of the Greek population sample (Table 9).

**Table 9:** The outcomes of the MANOVA test with the two factor-between-subject effects

Landmarks	Age	Sex	Age*Sex
Sg	0.18	0.26	0.07
G	0.27	0.55	0.14
N	0.57	0.98	0.30
Mn	0.64	0.48	0.42
Rhi	0.30	0.65	0.62
FeL	0.78	0.45	0.86
FeR	0.87	0.64	0.79
SoL	0.87	0.63	0.87
SoR	0.97	0.78	0.88
SkL	0.90	0.67	0.78
SkR	0.69	0.68	0.66
OrL	0.01**	0.03*	0.00**
OrR	0.16	0.27	0.04*
SmcL	0.12	0.37	0.14
SmcR	0.08	0.32	0.05*
FtL	0.36	0.46	0.10
FtR	0.56	0.66	0.29
FmtL	0.90	0.63	0.78
FmtR	0.86	0.58	0.53
EcL	0.93	0.63	0.94
EcR	0.85	0.99	0.39
MzL	0.04*	0.52	0.24
MzR	0.18	0.75	0.20
ZmL	0.33	0.52	0.21
ZmR	0.10	0.04*	0.41
JuL	0.41	0.73	0.84
JuR	0.21	0.83	0.47
ZyL	0.17	0.52	0.64
ZyR	0.13	0.38	0.35
CoL	0.30	0.15	0.26
CoR	0.02*	0.01	0.14
AlL	0.94	0.70	0.65
AlR	0.43	0.09	0.35
MnmL	0.37	0.41	0.59
MnmR	0.14	0.08	0.36
NmL	0.60	0.52	0.91
NmR	0.52	0.08	0.25
FmoL	0.95	0.64	0.66
FmoR	0.95	0.76	0.37

### Bilateral asymmetry

The comparison between the landmark pairs is summarised in Table 10. The FSTDs measured from the right side of the face were slightly larger than the left side in males. The greatest difference was measured between the left and right zygomaxillare (1.89 mm). Only the bilateral differences between the frontomalare temporale, zygomaxillare, mid-nasomaxillare, and nasomaxillare were statistically significant for males (*p* < 0.05).

In females, the left side of the face was larger than the right side. While the maximum depth difference was reported between the left and right zygomaxillare (1.14 mm), the minimum difference was recorded between the left and right frontal eminence, ectoconchion, and zygion (0.10 mm) (Table 10). The number of cranial landmarks that showed statistically significant bilateral asymmetry was higher in females than in males. The absolute mean depth differences in both males and females suggested that the bilateral asymmetry calculated between the landmark pairs was negligible.

**Table 10:** Paired sample t-test and Wilcoxon signed-rank test, comparing the FSTD values between the left and right sides of the face

Landmarks	Male		Female	
	Diff.	Sig.	Diff.	Sig.
FeL/FeR	0.07	0.20	0.10	0.62
SoL/SoR	0.04	0.68	0.37	0.32
SkL/SkR	0.08	0.30	0.42	0.27
OrL/OrR	0.22	0.10	0.18	0.99
SmcL/SmcR	1.21	0.07	0.57	0.45
FtL/FtR	0.13	0.22	0.13	0.25
FmtL/FmtR	0.57	0.02*	0.64	0.00**
EcL/EcR	0.06	0.55	0.10	0.29
MzL/MzR	0.55	0.43	0.43	0.03*
ZmL/ZmR	1.89	0.00**	1.14	0.00**
JuL/JuR	0.23	0.21	0.19	0.01**
ZyL/ZyR	0.39	0.06	0.10	0.02*
CoL/CoR	0.10	0.85	0.29	0.05*
AlL/AlR	0.20	0.44	0.32	0.85
MnmL/MnmR	0.10	0.05*	0.23	0.85
NmL/NmR	0.09	0.00**	0.21	0.39
FmoL/FmoR	0.13	0.60	0.14	0.12

Statistically significant at level * *p* < 0.05 and ** *p* < 0.01

### Interpopulation variation

The FSTDs of the Greek population were compared with the reported mean FSTDs of five populations (Tables 11, 12, 13, 14 and 15). The mean facial depth values of Greek males were slightly larger than those of the Cretan males, ranging between 0.06 and 2.04 mm. The highest depth difference was reported from the nasomaxillare. The FSTD differences between the supraglabella, mid-nasal, rhinion, supraorbital, orbitale, frontomalare temporale, ectoconchion, mid-zygomatic, alare, mid-nasomaxillare and nasomaxillare were at a statistically significant level (*p* < 0.05). The mean FSTD scores of the Cretan females were slightly lower than those of the Greek females at most of the landmarks (Table 11). The largest mean depth difference was calculated between the ectoconchion (1.86 mm). The smallest difference, however, was reported from the rhinion (0.02 mm). Apart from the nasion, rhinion, zygomaxillare and frontomalare orbitale, the *p*-values of all the landmarks showed statistically significant differences (*p* < 0.05).

The FSTD of the Greek males were negligible larger than those of the Turkish males. The highest depth difference was recorded at the supraorbital with 2.81 mm (Table 12). In contrast, the smallest difference was reported from the glabella with 0.12 mm. Apart from the glabella, all the facial depth values of the Greek males were found to be statistically different from those of the Turkish males. Similarly, the mean FSTD values of Greek females were slightly larger than those of the Turkish females at all the landmarks, apart from the nasion. The mean FSTD values of the nasion were 0.40 mm larger in the Turkish females. The mean value of the glabella was measured the same in both populations, which was 6.03 mm. This comparison showed that the mean FSTDs of Greek females were statistically different from those of Turkish females at the eight landmarks (*p* < 0.05). Only the *p-*value of the glabella was above 0.05 (*p* > 0.05).

Similar results were obtained from the comparison made between the Greek and Korean male subjects. The mean FSTD values of Greek males were larger than those of the Korean males. The results of one sample *t*-test showed that all the landmarks showed statistically significant differences at the significance level of *p* < 0.01, except the frontal eminence (Table 13). The mean differences between Greek and Korean females were compatible with those of males. The mean depth values of the Korean females were generally smaller than those of the Greek females. The largest variance was reported from the supraorbital (2.00 mm), which was a still negligible difference. All the *p*-values of landmarks, apart from the frontal eminence and mid-zygomatic, were below 0.05.

The comparison between the Czech and Greek males indicated that the FSTD values were higher in the Czech adult males than the Greek males at most of the landmarks. The mean depth differences of males varied between 0.01 mm and 4.82 mm. The highest depth differences were measured at the left and right ectoconchion (4.82 mm and 4.77 mm respectively) (Table 14). The *p*-values of nasion, left and right orbitale, left and right ectoconchion, right jugale, and left and right alare were lower than 0.05, indicating that the mean difference at these landmarks was statistically significant. On the contrary of the mean male depth values, the mean FSTD scores were larger in the Greek females than the Czech females. The largest depth differences were measured at the left and right ectoconchion (3.81 mm and 3.69 mm respectively) in females as well. Apart from the glabella, rhinion, left zygion, and left and right jugale, all the landmarks showed statistically significant differences (*p* < 0.01 and 0.05).

The FSTD datasets of male and female subjects were not reported separately for the French population sample [17]. Therefore, the comparison across the Greek and French subjects was made using the average depth values of all subjects. The French subjects showed the larger FSTD scores at most landmarks (Table 15). However, the mean depth differences were not significant and did not exceed 2.58 mm. Of 16 landmarks, the *p-*values of 13 landmarks were statistically significant, with a significance level of *p* < 0.01 and *p* < 0.05.

**Table 11:** Comparison of the mean FSTD values of the present study with the Cretan [37] population for male and female subjects

Landmarks	Male					Female				
	n	Greek	n	Cretan	Sig	n	Greek	n	Cretan	Sig
Sg	50	6.54	32	5.7	0.00**	48	5.93	32	4.8	0.00**
G	49	6.28	32	5.9	0.06	49	6.03	32	5.5	0.00**
N	50	8.02	32	7.8	0.35	50	6.76	32	6.7	0.76
Mn	41	3.30	30	4.5	0.00**	44	2.57	29	3.0	0.00**
Rhi	42	3.30	30	3.8	0.00**	44	2.62	24	2.6	0.70
Fe	45	6.27	32	6.1	0.42	49	5.55	32	3.9	0.00**
So	49	9.75	32	8.6	0.00**	50	8.40	32	7.2	0.00**
Sc	47	10.04	32	10.4	0.14	49	8.52	32	9.3	0.00**
Or	50	7.41	30	9.1	0.00**	49	6.52	24	7.4	0.00**
Smc	39	29.12	20	29.9	0.19	36	28.54	10	27.3	0.01**
Ft	48	6.19	32	6.4	0.32	50	5.77	32	4.6	0.00**
Fmt	48	7.91	32	7.0	0.00**	50	7.61	32	6.0	0.00**
Ec	45	10.61	32	9.4	0.03*	50	9.56	29	7.7	0.00**
Mz	41	9.49	24	11.2	0.00**	46	10.39	19	11.4	0.00**
Zm	36	15.75	20	16.6	0.28	44	15.38	13	14.8	0.14
Ju	48	9.66	32	9.6	0.84	50	10.62	31	9.9	0.00**
Zy	49	9.11	32	9.7	0.09	50	9.89	31	9.0	0.01**
Al	39	9.75	21	10.9	0.00**	42	9.23	10	10.0	0.02*
Mnm	45	3.24	30	2.9	0.00**	50	3.00	27	2.1	0.00**
Nm	44	6.04	29	4	0.00**	33	4.20	20	2.8	0.00**
Fmo	49	11.09	32	10.2	0.10	50	10.04	32	10.1	0.91

**Table 12:** Comparison of the mean FSTD values of the present study with the Turkish [22] population for male and female subjects

Landmarks	Male					Female				
	n	Greek	n	Turkish	Sig.	n	Greek	n	Turkish	Sig.
Sg	50	6.54	42	4.21	0.00**	48	5.93	42	3.94	0.00**
G	49	6.28	42	6.40	0.53	49	6.03	42	6.03	0.99
N	50	8.02	42	7.32	0.00**	50	6.76	42	7.16	0.03*
Rhi	42	3.30	42	2.97	0.00**	44	2.62	42	2.38	0.00**
Fe	45	6.27	42	4.48	0.00**	49	5.55	42	4.02	0.00**
So	49	9.75	42	6.94	0.00**	50	8.40	42	6.17	0.00**
Or	50	7.41	42	5.92	0.00**	49	6.52	42	5.47	0.00**
Mz	41	9.49	42	7.90	0.00**	46	10.39	42	9.40	0.00**
Zy	49	9.11	42	7.83	0.00**	50	9.89	42	8.06	0.00**

**Table 13:** Comparison of the mean FSTD values of the present study with the Korean [23] population for male and female subjects

Landmarks	Male					Female				
	n	Greek	n	Korean	Sig.	n	Greek	n	Korean	Sig.
Sg	50	6.54	50	5.3	0.00**	48	5.93	50	4.8	0.00**
G	49	6.28	50	5.6	0.00**	49	6.03	50	5.3	0.00**
N	50	8.02	50	6.4	0.00**	50	6.76	50	5.4	0.00**
Rhi	42	3.30	50	2.3	0.00**	44	2.62	50	2.2	0.00**
Fe	45	6.27	50	6.2	0.70	49	5.55	50	5.4	0.39
So	49	9.75	50	7.2	0.00**	50	8.40	50	6.4	0.00**
Mz	41	9.49	50	8.6	0.00**	46	10.39	50	10.2	0.41
Zy	49	9.11	50	8.1	0.00**	50	9.89	50	8.7	0.00**

**Table 14:** Comparison of the mean FSTD values of the present study with the Czech [19] population for male and female subjects

Landmarks	Male					Female				
	n	Greek	n	Czech	Sig.	n	Greek	n	Czech	Sig.
G	49	6.28	56	6.29	0.94	49	6.03	46	6.01	0.80
N	50	8.02	56	9.41	0.00**	50	6.76	46	8.25	0.00**
Rhi	42	3.30	56	3.11	0.10	44	2.62	46	2.61	0.81
OrL	50	7.18	56	8.37	0.00**	47	6.34	46	7.47	0.00**
OrR	50	7.41	56	8.35	0.01**	49	6.52	46	7.22	0.01**
EcL	49	10.67	56	5.85	0.00**	48	9.66	46	5.85	0.00**
EcR	45	10.61	56	5.84	0.00**	50	9.56	46	5.87	0.00**
ZyL	49	8.71	56	8.77	0.85	49	9.79	46	9.19	0.09
ZyR	49	9.11	56	8.49	0.08	50	9.89	46	9.17	0.05*
JuL	49	9.42	56	9.72	0.30	49	10.43	46	10.27	0.56
JuR	48	9.65	56	10.36	0.00**	50	10.62	46	10.56	0.82
AlL	42	9.95	56	12.35	0.00**	43	9.55	46	10.69	0.00**
AlR	39	9.75	56	12.02	0.00**	42	9.23	46	10.62	0.00**

**Table 15:** Comparison of the mean FSTD values of the present study with the French [17] population

Landmarks	*n*	Greek	*n*	French	Sig.
G	98	6.16	366	6.5	0.00**
N	100	7.39	469	8.2	0.00**
Mn	85	2.92	321	5.5	0.00**
Rhi	86	2.96	459	3.0	0.54
Sc	96	9.26	373	9.7	0.03*
Or	99	6.97	371	8.6	0.00**
Smc	75	28.84	330	28.2	0.08
Ft	98	5.98	361	6.7	0.00**
Fmt	98	7.76	242	9.1	0.00**
Ec	95	10.06	372	7.8	0.00**
Zm	80	15.55	353	15.1	0.09
Ju	98	10.15	245	10.9	0.00**
Zy	99	9.50	364	10.0	0.04*
Co	99	17.73	215	16.7	0.02*
Mnm	95	3.11	328	3.5	0.00**
Nm	77	5.25	457	4.9	0.04*

## Discussion

### Accuracy of head and skull segmentations

Following the Daubert v. Merrell Dow Pharmaceuticals, Inc. [58] case, new standards for scientific evidence were introduced in forensic disciplines. To be considered scientifically valid, evidence must be testable, reproducible with a known error rate, subject to peer review, and accepted by the respective scientific community [39]. These admissibility criteria led many forensic disciplines to question the validity of their existing methods. Forensic anthropology was one such field that sought to establish standards. However, there are still aspects of the discipline, such as estimating age and approximating faces, that rely on subjective interpretation. To address this issue and improve the reliability of the methods, Christensen and Crowder [40] recommended reporting the repeatability of the methods used.

The study found that the 3D head and skull models were accurately reproduced using the Histogram method. This assessment was crucial in ensuring the precision of the re-segmentations prior to the depth measurements. The results of this study have significant implications, as the Histogram method can be applied to other fields that require the segmentation of CT images. In FSTD studies, the half maximum height (HMH) protocol is typically used for segmentation. However, this method is time-consuming as it requires a lengthy calculation process of HMH values for each individual, as well as an additional edge correction. On the other hand, the Histogram method also requires edge correction when necessary but is more time-efficient as it does not require quantifying HMH values.

### Intra- and inter-observer error

Some degree of measurement error is always expected from the studies in which anthropometric measurements are involved [32]. In the FSTD literature, the technical error of the measurements is rarely reported. Stephan et al. [41] suggested calculating TEM and rTEM values to increase the integrity between the FSTD studies. In this study, the facial soft tissue depth measurements were tested for repeatability and reliability within and between observers. The results of the TEM, rTEM and R scores of the intra-observer error were within the acceptable level of accuracy.

**Fig. 5:**
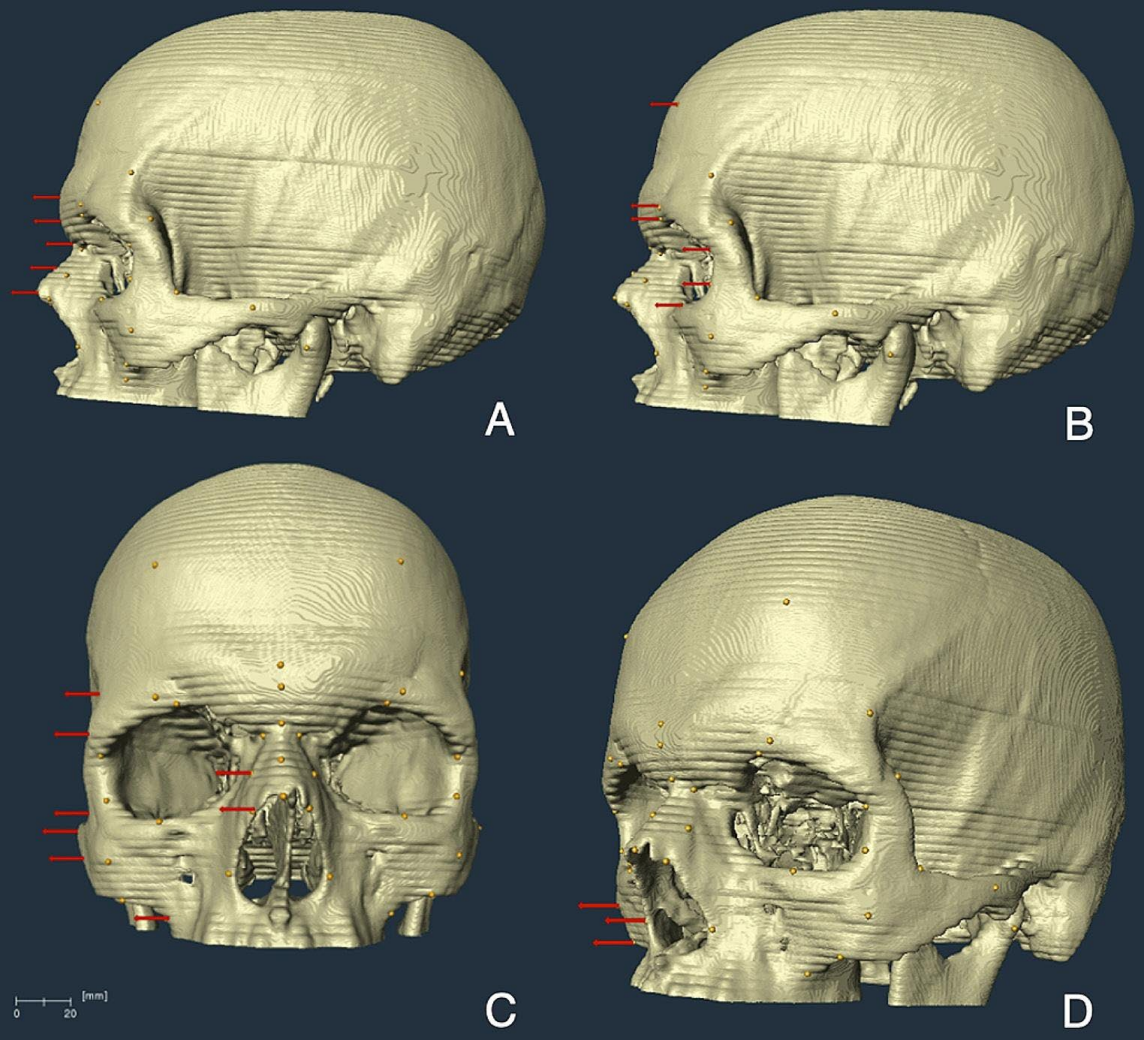
The measurement directions of the landmarks. Mid-sagittal **(A)** and a number of bilateral **(B)** landmarks were measured when the skull was in the sagittal plane. The rest of the landmarks were measured when the skull was in the coronal **(C)** and/or sagitto-coronal **(D)** planes

All the measurements carried out were subject to skull positioning-associated errors. Since the measurements were taken from the 3 different planes (Fig. 5), the skulls were rotated, and their positions were re-adapted each time based on the location of landmarks. This methodological requirement might have led to a divergence between the repeating measurements.

Bookstein [42] divides landmarks into three types based on their anatomical shape and position. Type I landmarks are described as discrete juxtapositions of tissues. These landmarks are found where the three structures and/or sutures meet, such as the nasion, nasomaxillofrontale, nasomaxillare and zygomaxillare. Type II landmarks are defined as maxima of curvature or local morphometric processes, involving the tips of the processes, and valleys of invaginations such as the jugale, supraglabella, rhinion and ectoconchion [43]. Type III landmarks are, however, defined as those found at the extremal locations, such as the glabella, frontomalare orbitale, frontotemporale, zygion, and sub-maxillar curvature. Studies showed that Type I landmarks are identified with high accuracy, whereas Type II and III landmarks are prone to misidentification [38, 43–45]. Despite these findings, the rTEMs of the zygomaxillare, mid-nasomaxillare and nasomaxillare (Type I landmarks) of this study appeared to be high. This result could be explained by the failure of reconstruction of the zygomaticomaxillary and nasomaxillary sutures. This issue might have caused inconsistent identification of the respective landmarks on these sutures. Bertoglio et al. [47] stated that the non-metric traits and facial sutures, which are formed by the thin layer of compact bone, are less likely to be visible on 3D models (especially maxillary bone). Additionally, Kim et al. [46] found that the quality of images decreases with increasing slice thickness, causing less precise 3D models. Accordingly, the images with the 0.5 mm (and below) slice thickness provide the best image quality [46]. Although the CT slice thickness of this study (2.4 mm) was under 5 mm, it could be still considered thick to recreate the facial sutures accurately when their size is taken into account.

### Influence of age and sex on FSTD

The impact of age on FSTDs has been studied by several studies, which have reported varying results. Wilkinson [14] revisited previously published FSTD studies and concluded that the influence of age on the FSTD was variable. As the general tendency suggests, the depth at the mouth and lower cheek region decreases, whereas the depth at the chin and brow region increases with age [14, 59]. Both increasing and decreasing trends are expected from the facial depth since the volume of the superficial and deep facial fat compartments change with age [52, 53].

In this study, the greatest depth values of males were mainly reported from the 35–44 years age group. Yet, after this age group, the mean values did not display any regular pattern, they fluctuated. Therefore, it was not possible to relate the mean facial depth of any landmarks and/or facial regions (e.g., cheek, orbital, nasal) with increasing age. This fluctuation might be attributed to the varying body weight of the individuals measured for this study. The impact of body weight on FSTD has been analysed by several researchers [26, 49, 51]. Although there is a slight deviation across the results of the studies, the general agreement is that FSTD increases as body weight increases. Since the individuals in this study could not be categorised based on their BMIs, the age groups of both sex categories might have consisted of subjects with mixed body types (e.g., slender, normal and overweight). This issue might have obscured the real impact of age and sex variables on the FSTD. In females, the FSTD values increased with age at the nasal, orbital and cheek regions. The depth values mostly peaked at the 55–64 years age group, then decreased.

The researchers have investigated the influence of sex on FSTD and found that males have larger facial depth than females [20, 23, 26, 27]. In particular, the brow, jaw and mouth regions are thicker in males, whereas the cheek region is thicker in females [14, 26, 27]. The findings of the current study also demonstrated that male depth values were generally higher than those of female depth values.

Cha [54] found some depth differences statistically significant between sexes, yet not exceeding 2 mm. This difference was attributed to the facial fat compartments, as well as the age-dependent decrease in skin thickness. In general, the skin thickness appears to be larger in males than in females. With increasing age, the skin undergoes thinning, dryness and rough texture. In addition, the density of collagen in the skin, which is the main contributor to skin thickness, decreases with advancing age [55]. This decrease occurs at different rates in males and females [56]. While male skin demonstrates progressive thinning with ageing, female skin remains steady until around middle age, and then rapid alteration occurs [56, 57]. In this study, the mean FSTDs of males showed inconsistently increasing and decreasing trends between the age groups. Therefore, there was not enough evidence to relate the depth changes to a possible impact of skin thinning in males. The female depth scores, on the other hand, decreased after the 55–64 years age group. The decrease in mean FSTDs of females might be explained by skin thinning along with the decrease in the volume of the facial fat masses and attenuation of the facial soft tissues.

Although the depth differences between sexes were reported as statistically significant by some scholars, the depth variation was reported as insignificant [17, 25, 28]. As a result, they recommend combining depth measurements to create a general population database, as sex has little impact on facial soft tissue depths. This study’s findings are in agreement with these previous studies [17, 28], showing that the differences in depth between landmarks were mostly insignificant. Furthermore, the variation in depth within and across sex categories was low. Therefore, this study supports the idea put forward by Stephan and Simpson [25] that a general dataset can be generated for both males and females since sex has no significant impact on facial soft tissue depth in this population sample.

### Bilateral asymmetry

Contrary to the general belief, the human body rarely displays complete symmetry. The degree of asymmetry may vary, and it may not always be evident to the naked eye [48]. The assessment of facial asymmetry showed variety across the FSTD studies, yet this factor was not taken into account in all studies. The general agreement in the FSTD literature regarding facial asymmetry is that statistically significant variation between the left and right sides of the face might be observed at varying numbers of landmarks, yet the absolute mean differences between the bilateral landmarks are insignificant [23, 25, 49]. Therefore, researchers either averaged bilateral depth values and reported one single value for each bilateral landmark [25, 50], or measurements were taken only from the left side of the face by anthropological convention [16, 21]. Alternatively, although the landmarks at both sides were measured, only the right side of the face was reported since the absolute mean depth differences were insignificant [18, 37, 49].

In this study, the right side of the face was found to be slightly thicker in males than the left side. In females, however, the left side was insignificantly larger than the right side. This bilateral variation appeared to be small. Thus, measuring one side of the face and using the FSTD dataset of only one side would not compromise facial approximations.

### Interpopulation variation

Researchers reported varied inter-population depth results over time. Some studies suggested establishing population-specific datasets since significant depth variations were acquired across the studies [21, 26, 37], whereas other studies claimed that there are negligible differences between the datasets. These differences have little practical significance [16, 17, 20, 28].

An extensive FSTD comparison was made by Stephan and Simpson [28] by using the datasets of 55 populations. As they reported, the FSTD scores varied across the populations. However, they also reported that variations within the populations were greater than the variations across the populations. The difference obtained cannot be attributed only to the inter-population difference since all the studies investigated used different data-obtaining methods (e.g., ultrasound, radiograph, MRI, CT, CBCT), measurement methods (i.e., measurements from cadavers, measurements from 2D images and measurements from 3D models), and the FSTDs were measured by different examiners. Stephan and Simpson [28] concluded that inter-population FSTD differences are of minor practical importance for facial approximation. Therefore, all the population datasets should be pooled in order to increase the sample size and establish one statistically powerful dataset for all populations.

Although there were statistically significant differences in the facial depth at most landmarks, the variation in depth across populations was minimal for both males and females in this study. This result was in accordance with the results of other studies [16, 17, 24, 28]. The mean depth differences obtained from geographically close and distant populations to the Greek population did not vary considerably. This suggests that there is no positive correlation between geographical distance and facial depth. Although the population comparison of this study was made between the studies that used similar data-obtaining methods and measurement protocols, the impact of the examiner and individual facial depth variation should be taken into account. As suggested by Stephan and Simpson [28], pooling the FSTD datasets of all other populations might generate a single, comprehensive, and statistically powerful dataset.

## Conclusion

The facial soft tissue dataset is a significant guideline for facial approximation and superimposition methods [25, 28]. Although the results of a number of other studies [17, 20, 24, 28] reported small inter-population variations, researchers are still encouraged to establish a population-specific dataset so that a pooled dataset could be built [28]. The result of this study contributes to the craniofacial approximation literature by compiling the FSTD dataset for the Greek population for the first time.

The database generated in this study is significant not only for the identification of unknown Greek individuals but also for a broader perspective, it is important to contribute literature to create a more comprehensive dataset that can be used for all populations. The identification of the victims of migration, natural disasters, crime and armed conflicts is a significant problem due to the lack of antemortem data. In that case, performing forensic anthropological methods and facial approximation of victims might assist in identifying the remains. When population affinity cannot be accurately set, a weighted FSTD database could be used.

## Data Availability

The datasets generated during the current study are available from the corresponding author on reasonable request.

## References

[1] İşcan MY, Kennedy KAR (1989) Reconstruction of life from the skeleton. Alan R. Liss Inc, New York, pp 1–10

[2] Cattaneo C (2007) Forensic anthropology: developments of a classical discipline in the new millennium. Forensic Sci Int 165:185–193. 10.1016/j.forsciint.2006.05.018 16843626 10.1016/j.forsciint.2006.05.018

[3] Dirkmaat DC (2012) A companion to forensic anthropology. First Edition. Blackwell Publishing, Sussex, pp 43–47

[4] Scheuer L, Black S (2007) Osteology. In: Thompson T, Black S (eds) Forensic human identification an introduction. CRC, Boca Raton, pp 199–220

[5] Brough AL, Morgan B, Rutty GN (2015) The basics of disaster victim identification. J Forensic Rad Imaging 3:29–37. 10.1016/j.jofri.2015.01.002

[6] Goodwin W (2017) The use of forensic DNA analysis in humanitarian forensic action: the development of a set of international standards. Forensic Sci Int 278:221–227. 10.1016/j.forsciint.2017.07.002 28755626 10.1016/j.forsciint.2017.07.002

[7] Wilkinson C (2007) Facial anthropology and reconstruction. In: Thompson T, Black S (eds) Forensic human identification an introduction. CRC, Boca Raton, pp 231–256

[8] Jensen RA (1999) Mass fatality and casualty incidents: a field guide. CRC, Bota Racon

[9] Christensen AM, Anderson BE (2017) Methods of personal identification. In: Langley NR, Tersigni-Tarrant MA (Eds) Forensic anthropology: a comprehensive introduction. Second Edition. Boca Raton: CRC Press, pp. 313–333

[10] Evison MP (2009) Forensic anthropology and human identification from the skeleton. In: Fraser J, Williams R (eds) Handbook of forensic science. Devon, Willian Publishing, pp 85–112

[11] İşcan MY, Steyn M (2013) Facial approximation and skull-photo superimposition. In: İşcan MY, Steyn M (Eds) The human skeleton in forensic medicine. Third Edition. Springfield: Charles C Thomas Publisher, pp. 361–392

[12] Sauer NJ, Michael AR, Fenton TW (2012) Human identification using skull-photo superimposition and forensic image comparison. In: Dirkmaat DC (Ed) A companion to forensic anthropology. First Edition. West Sussex, Wiley-Blackwell Publication, pp. 432–446

[13] Taylor KT (2001) Forensic art and illustration. CRC, Boca Raton, pp 361–417

[14] Wilkinson C (2004) Forensic facial reconstruction. Cambridge University Press, Cambridge, pp 157–199

[15] Chung JH, Chen HT, Hsu WY, Huang GS, Shaw KP (2015) A CT scan database for the facial soft tissue thickness of Taiwan adults. Forensic Sci Int 253:132e1–e11. 10.1016/j.forsciint.2015.04.028 10.1016/j.forsciint.2015.04.028 26028278

[16] Dong Y, Huang L, Feng Z, Bai S, Wu G, Zhao Y (2012) Influence of sex and body mass index on facial soft tissue thickness measurements of the northern Chinese adult population. Forensic Sci Int 222:396e1–396e7. 10.1016/j.forsciint.2012.06.004 10.1016/j.forsciint.2012.06.004 22738738

[17] Guyomarc’h P, Santos F, Dutailly B, Coqueugniot H (2013) Facial soft tissue depth in French adults: variability, specificity and estimation. Forensic Sci Int 231:411e1–411e10. 10.1016/j.forsciint.2013.04.007 10.1016/j.forsciint.2013.04.007 23684263

[18] Panenková P, Benus R, Masnicova S, Obertova Z, Grunt J (2012) Facial soft tissue thicknesses of the mid-face for Slovak population. Forensic Sci Int 220:293e1–293e6. 10.1016/j.forsciint.2012.02.015 10.1016/j.forsciint.2012.02.015 22430009

[19] Drgáčová A, Dupej J, Veleminska J (2016) Facial soft tissue thicknesses in the present Czech population. Forensic Sci Int 260:e101–e107. 10.1016/j.forsciint.2016.01.011 10.1016/j.forsciint.2016.01.011 26860069

[20] Thiemann N, Keil V, Roy U (2017) In vivo facial soft tissue depth of a modern adult population from Germany. Int J Legal Med 131:1455–1488. 10.1007/s00414-017-1581-y 28417258 10.1007/s00414-017-1581-y

[21] Cavanagh D, Steyn M (2011) Facial reconstruction: soft tissue thickness values for South African black females. Forensic Sci Int 206. 10.1016/j.forsciint.2011.01.009. 215.e1–215.e7 10.1016/j.forsciint.2011.01.009 21288672

[22] Bulut O, Sipahioglu S, Hekimoglu B (2014) Facial soft tissue thickness database for craniofacial reconstruction in the Turkish adult population. Forensic Sci Int 242:44–61. 10.1016/j.forsciint.2014.06.012 25023216 10.1016/j.forsciint.2014.06.012

[23] Hwang HS, Park MK, Lee WJ, Cho JH, Kim BK, Wilkinson C (2012) Facial soft tissue thickness database for craniofacial reconstruction in Korean adults. J Forensic Sci 57(6):1442–1447. 10.1111/j.1556-4029.2012.02192.x 22621203 10.1111/j.1556-4029.2012.02192.x

[24] Sahni D, Sanjeev, Singh G, Jit I, Singh P (2008) Facial soft tissue thickness in Northwest Indian adults. Forensic Sci Int 176:137–146. 10.1016/j.forsciint.2007.07.012 17997243 10.1016/j.forsciint.2007.07.012

[25] Domaracki M, Stephan CN (2006) Facial soft tissue thicknesses in Australian adult cadavers. J Forensic Sci 51(1):5–10. 10.1111/j.1556-4029.2005.00009.x 16423216 10.1111/j.1556-4029.2005.00009.x

[26] Codinha S (2009) Facial soft tissue thicknesses for the Portuguese adult population. Forensic Sci Int 184:80e1–8080. .e7 10.1016/j.forsciint.2008.11.011 19124207

[27] De Greef S, Vandermeulen D, Claes P, Suetens P, Williems G (2009) The influence of sex, age and body mass index on facial soft tissue depth. Forensic Sci Med Pathol 5:60–65. 10.1007/s12024-009-9085-9 19437147 10.1007/s12024-009-9085-9

[28] Stephan CN, Simpson EK (2008) Facial soft tissue depth in craniofacial identification (part I): an analytical review of the published adult data. J Forensic Sci 53(6):1257–1272. 10.1111/j.1556-4029.2008.00852.x 18783476 10.1111/j.1556-4029.2008.00852.x

[29] Karell AM, Langstaff HK, Halazonetis DJ, Minghetti C, Frelat M, Kranioti EF (2016) A novel method for pair-matching using three-dimensional digital models of bone: mesh-to-mesh value comparison. Int J Legal Med 130:1315–1322. 10.1007/s00414-016-1334-3 26966098 10.1007/s00414-016-1334-3 PMC4976056

[30] Garson JG (1885) The Frankfort craniometric agreement with critical remarks thereon. J Anthropol Inst Great Brit Irel 14:64–83. 10.2307/2841484

[31] Aspert N, Santa-Cruz D, Ebrahimi T (2002) Mesh: measuring errors between surfaces using the Hausdorff distance. Proc IEEE Int Conf Multimedia Expo 1:705–708. 10.1109/ICME.2002.1035879

[32] Jamaiyah H, Geeta A, Safiza MN, Khor GL, Wong NF, Kee CC, Rahmah R, Ahmad AZ, Suzana S, Chen WS, Rajaah M, Adam B (2010) Reliability, technical error of measurements and validity of length and weight measurements for children under two years old in malasia. Med J Malaysia 65(A):131–137 21488474

[33] Perini TA, De Oliveira GL, Dos Santos Ornellas J, De Oliveira FP (2005) Technical error of measurement in anthropometry. Rev Bras Med Esporte 11(1):86–90. 10.1590/S1517-86922005000100009

[34] Ulijaszek SJ, Kerr DA (1999) Anthropometric measurement error and the assessment of nutritional status. Br J Nutr 82:165–177. 10.1017/S0007114599001348 10655963 10.1017/s0007114599001348

[35] Stephen JM, Calder JDF, Williams A, Daou HE (2021) Comparative accuracy of lower limb bone geometry determined using MRI, CT, and direct bone 3D models. J Orthop Res 39:1870–1876. 10.1002/jor.24923 33222265 10.1002/jor.24923

[36] Ballester MAG, Zisserman AP, Brady M (2002) Estimation of the partial volume effect in MRI. Med Image Anal 6(4):389–405. 10.1016/S1361-8415(02)00061-0 12494949 10.1016/s1361-8415(02)00061-0

[37] Somos CP, Rea PM, Shankland S, Kranioti EF (2019) Medical imaging and facial soft tissue thickness studies for forensic craniofacial approximation: a pilot study on modern cretans. In: Rea PM (ed) Biomedical Visualisation, advances in Experimental Medicine and Biology, volume II, 1138. First Edition. Springer, Switzerland. 10.1007/978-3-030-14227-8_6 10.1007/978-3-030-14227-8_6 31313259

[38] Guyomarc’h P, Santos F, Dutailly B, Desbarats P, Bou C, Coqueugniot H (2012) Three-dimensional computer-assisted craniometrics: a comparison of the uncertainty in measurement induced by surface reconstruction performed by two computer programs. Forensic Sci Int 219:221–227. 10.1016/j.forsciint.2012.01.008 22297143 10.1016/j.forsciint.2012.01.008

[39] Grivas CR, Komar DA (2008) Kumho, Daubert, and the nature of scientific inquiry: implications for forensic anthropology. J Forensic Sci 53(4):771–776. 10.1111/j.1556-4029.2008.00771.x 18489550 10.1111/j.1556-4029.2008.00771.x

[40] Christensen AM, Crowder CM (2009) Evidentiary standards for forensic anthropology. J Forensic Sci 54(6):1211–1216. 10.1111/j.1556-4029.2009.01176.x 19804520 10.1111/j.1556-4029.2009.01176.x

[41] Stephan CN, Meikle B, Freudenstein N, Taylor R, Claes P (2019) Facial soft tissue thicknesses in craniofacial identification: data collection protocols and associated measurement errors. Forensic Sci Int 304:1–20. 10.1016/j.forsciint.2019.109965 10.1016/j.forsciint.2019.109965 31610333

[42] Bookstein FL (1991) Landmarks. In: Bookstein FL (ed) Morphometric Tools for Landmark Data. Cambridge University Press, Cambridge, pp 55–87

[43] Sholts SB, Flores L, Walker PL, Wärmländer SKTS (2011) Comparison of coordinate measurement precision of different landmark types on human crania using a 3D laser scanner and a 3D digitiser: implications for applications of digital morphometrics. Internat J Osteoarchaeol 21:535–543. 10.1002/oa.1156

[44] Ross AH, Williams SE (2008) Testing repeatability and error of coordinate landmark data acquired from crania. J Forensic Sci 53(4):782–785. 10.1111/j.1556-4029.2008.00751.x 18537868 10.1111/j.1556-4029.2008.00751.x

[45] Slice DE, Unteregger C, Bookstein FL, Schäfer K (2004) Modeling the precision of landmark data. Am J Phys Anthropol 123(s38):183–195

[46] Kim KD, Ruprecht A, Wang G, Lee JB, Dawson DV, Vannier MW (2005) Accuracy of facial soft tissue thickness measurements in personal computer-based multiplanar reconstructed computed tomographic images. Forensic Sci Int 155:28–34. 10.1016/j.forsciint.2004.11.004 16216709 10.1016/j.forsciint.2004.11.004

[47] Bertoglio B, Corradin S, Cappella A, Mazzarelli D, Biehler-Gomez L, Messina C, Pozzi G, Sconfienza LM, Sardanelli F, Sforza C, De Angelis D, Cattaneo C (2020) Pitfalls of computed tomography 3D reconstruction models in cranial nonmetric analysis. J Forensic Sci 65(6):2098–2107. 10.1111/1556-4029.14535 32809248 10.1111/1556-4029.14535

[48] Dangerfield PH (1994) Asymmetry and growth. In: Ulijaszek SJ, Mascie-Taylor CGN (eds) Anthropometry: the individual and the population. First Edition. Cambridge University Press, Cambridge, pp 7–29

[49] De Greef S, Claes P, Vandermeulen D, Mollemans W, Suetens P, Willems G (2006) Large scale in-vivo caucasian facial soft tissue thickness database for craniofacial reconstruction. Forensic Sci Int 159S:S126–S146. 10.1016/j.forsciint.2006.02.034 10.1016/j.forsciint.2006.02.034 16563680

[50] Simpson E, Henneberg M (2002) Variation of soft tissue thicknesses on the human face and their relation to craniometric dimensions. Am J Phys Anthropol 118:121–133. 10.1002/ajpa.10073 12012365 10.1002/ajpa.10073

[51] Sutton PRN (1969) Bizygomatic diameter: the thickness of the soft tissues over the zygions. Am J Phys Anthropol 30:303–310. 10.1002/ajpa.1330300215 5772049 10.1002/ajpa.1330300215

[52] Rohrich RJ, Pessa JE (2007) The fat compartments of the face: anatomy and clinical implications for cosmetic surgery. Plast Reconstr Surg 119(7):2219–2227. 10.1097/01.prs.0000265403.66886.54 17519724 10.1097/01.prs.0000265403.66886.54

[53] Donofrio LM (2000) Fat distribution: a morphological study of the aging face. Dermatol Surg 26(12):1107–1112. 10.1046/j.1524-4725.2000.00270.x 11134986

[54] Cha KS (2013) Soft-tissue thickness of South Korean adults with normal facial profiles. Korean J Orthod 43(4):178–185. 10.4041/kjod.2013.43.4.178 24015387 10.4041/kjod.2013.43.4.178 PMC3762959

[55] Shuster S, Black MM, McVitie E (1975) The influence of age and sex on skin thickness, skin collagen and density. Br J Dermatol 93:639–643. 10.1111/j.1365-2133.1975.tb05113.x 1220811 10.1111/j.1365-2133.1975.tb05113.x

[56] Tur E (1997) Physiology of the skin- differences between women and men. Clin Dermatol 15:5–16. 10.1016/S0738-081X(96)00105-8 9034651 10.1016/s0738-081x(96)00105-8

[57] Luebberding S, Krueger N, Kerscher M (2014) Mechanical properties of human skin in vivo: a comparative evaluation in 300 men and women. Skin Res Technol 20:127–135. 10.1111/srt.12094 23889488 10.1111/srt.12094

[58] Daubert v (1993) Merrell Dow Pharmaceuticals Inc 509 US 579

[59] De Greef S, Vandermeulen D, Claes P, Suetens P, Williems G (2009) The influence of sex, age and body mass index on facial soft tissue depth. Forensic Sci Med Pathol 5:60–65 19437147 10.1007/s12024-009-9085-9

